# Coccygeal Pad With an Anterior Flexed Coccyx: A Case Report

**Published:** 2016-08-31

**Authors:** Rina Hikiami, Natsuko Kakudo, Yuko Iguchi, Motoki Katsube, Naoki Morimoto, Masakatsu Hihara, Toshihito Mitsui, Takashi Yamauchi, Kenji Kusumoto

**Affiliations:** ^a^Department of Plastic and Reconstructive Surgery, Kansai Medical University, Osaka, Japan; ^b^Department of Plastic and Reconstructive Surgery, Graduate School of Medicine, Kyoto University, Kyoto, Japan

**Keywords:** coccygeal pad, anterior flex, sacrococcygeal angle, hyperkeratosis, epidermal hyperplasia

## Abstract

**Objective:** A coccygeal pad is a nodular lesion in the sacrococcygeal area. It is a rare disease characterized by corneal thickening, the proliferation of collagen bundles, and an anterior flexed coccyx. In the English literature, 42 patients have been reported. **Methods:** We present a young male case of a typical coccygeal pad with an anterior flexed coccyx. **Results:** After resection of the nodule and coccygectomy, there has been no recurrence during the 6-month postoperative follow-up. **Conclusions:** Plastic surgeons should recognize this as a disease to be differentiated from gluteal skin tumors.

A coccygeal pad is a nodular lesion in the sacrococcygeal area, accompanied by an anterior flexed coccyx. In the nodule, marked proliferation of collagen bundles with slight dermal edema is observed histopathologically. This nodule was first reported by Ota et al^1^ in Japan. Since then, in the English literature, 42 patients have been reported mainly in dermatological field. Of these, no case has been reported in the field of plastic surgery. To diagnose a coccygeal pad, it is necessary to differentiate this disease from human tail, pilonidal sinus, and coccygeal cysts. Till now, the pathogenesis has not been clarified.

In this report, we performed resection of the coccyx with anterior flex and a nodule in a young male patient to treat a coccygeal pad in the sacrococcygeal area. There has been no recurrence during the 6-month postoperative follow-up. Favorable results were obtained. We report the present case and review the literature on the diagnosis and treatment of this disease.

## METHODS

**Figure 1:**
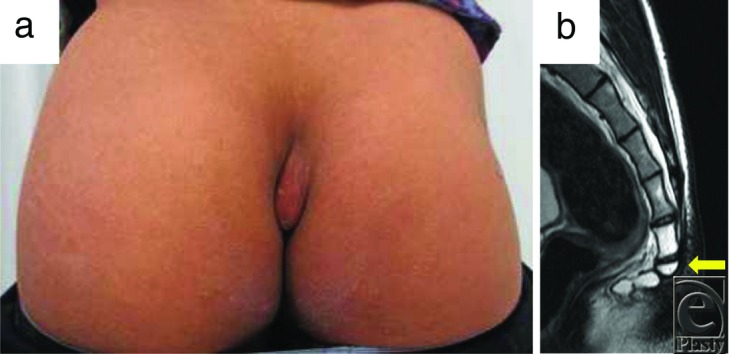
(a) Preoperative findings. (b) Preoperative magnetic resonance imaging (T2) findings. The arrow indicates anterior bending of the coccyx.

**Figure 2:**
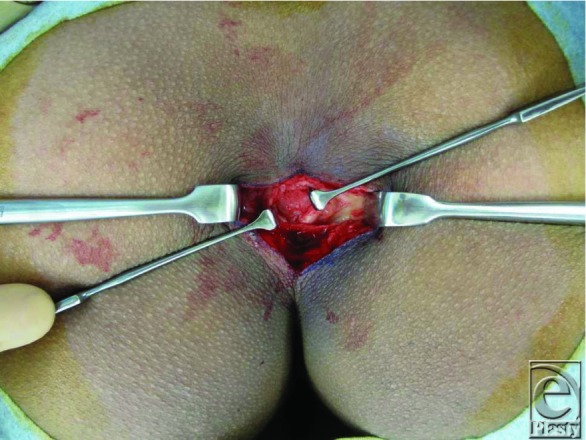
Intraoperative findings. The protruding coccyx was resected.

The patient was an 18-year-old man with a nodule in the coccygeal region. Ten years ago, he noticed the coccygeal nodule, and it has gradually enlarged. The elastic-hard nodule, 7 × 4 cm in diameter, was positioned cephalad in the gluteal cleft (Fig 1*a*). The nodule was an induration including overlayed skin and had no adhesion to the deep structures. In the magnetic resonance image, the nodular mass was well bordered with a low signal on T1 and T2 (Fig 1*b*). It was localized at the outer side of the second to fourth coccyges, and the sacrococcygeal joint was flexed anteriorly at the right angle (Fig 1*b*). The nodule was resected with a small overlayed skin, and the prominence of the sacrococcygeal joint was shaved and rounded using a filea rasp under general anesthesia (Fig 2). After hemostasis, bilateral wound margins were carefully buried, sutured with the anchor sutures against the sacrococcygeal periosteum. The skin sutures were kept for 12 days, a little longer than usual skin sutures. After removal of sutures, conservative therapy using sponge pressure with a skin tape was given for 3 months.

## RESULTS

**Figure 3:**
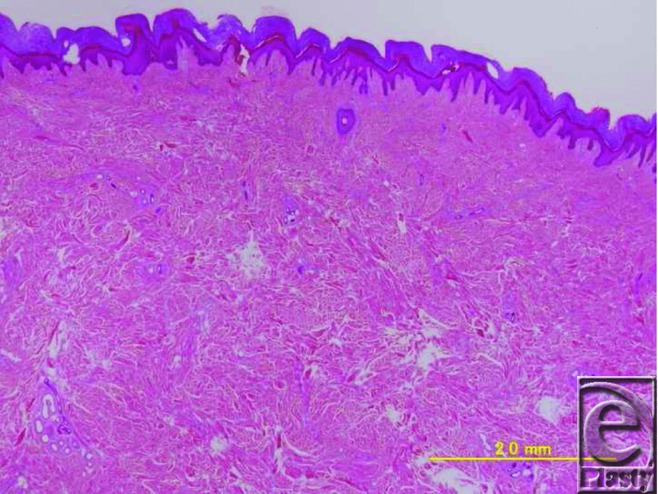
Histological findings of the excised nodule. Hyperkeratosis and proliferation of collagen bundles were observed (hematoxylin and eosin stain, bar: 20 mm).

**Figure 4:**
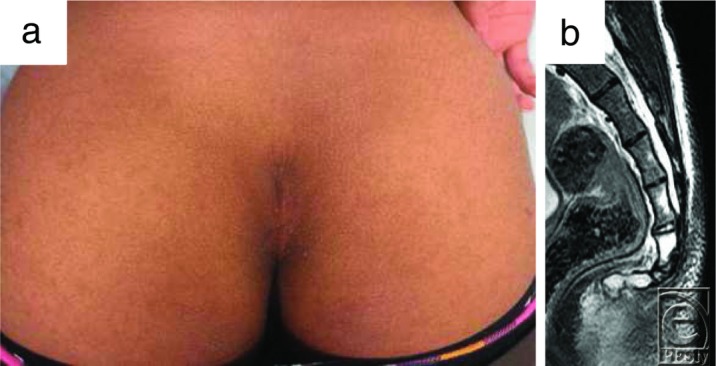
(a) Postoperative findings. (b) Postoperative magnetic resonance imaging (T2) findings.

Histopathological examination revealed mild epidermal hyperplasia and proliferation of collagen fibers and fibroblasts in the thick dermis layer (Fig 3). From its pathological findings, the final diagnosis was confirmed as a coccygeal pad. Six months postoperatively, no recurrent signs were shown at the operative site (Fig 4).

## DISCUSSION

The coccygeal pad is a protrusive skin lesion with slight keratinization involving the sacrococcygeal area. Histologically, it is characterized by a slight skin thickening and a proliferative thickening of corium collagen fibers.^2^ It shows a flexion deformity of the coccyx, and repeated stimulation of the coccygeal flexion site may cause protrusive skin lesions.^2^ This disease was presented as “tylosis-like eruption in the sacral area” by Ota et al in 1985.^1^ Since then, a few cases have been reported in Japan.^3^^,^^4^ In 1993, Murasawa et al^2^ named this disease “coccygeal pad,” based on the pathogenesis and histological findings of the lesions. Since then, the name “coccygeal pad” has commonly been used. Approximately 75 cases have been reported. However, only 42 of these were presented in the English literature because the disease is rare. Nodular lesions in the sacrococcygeal area, the so-called coccygeal pad, have not been reported in the English published work, except for reports from Japan.

According to statistics regarding the coccygeal pad based on a search of PubMed for the English-language published work,^5^ the ages ranged from 13 to 18 years in 67% of patients. The mean age was 19 years, and 81% of the patients were male. The present case was an 18-year-old man, being consistent with these profiles.

A study reported that anterior dislocation of the coccyx related to sacrococcygeal joint angles positioned sharply forward was observed in 80% of patients with coccygeal pads.^5^ In the present case, anterior flexion of a combination of the second, third, and fourth coccyges was also noted, and a nodule was observed immediately above the coccygeal protrusion site. This disease was suggestive of a bicycle saddle in some patients^6^ and with a relaxed sitting position on a hard school chair in others.^5^ Furthermore, some patients developed this disease without anterior flexion of the coccyx; the cause remains to be clarified.

In regard to clinical appearance, 74% of the patients were asymptomatic whereas 26% of them complained of tenderness.^5^ In the English published work, pain affecting coccyx is referred to as coccydynia.^7^ The precise cause is uncertain, but many authors have focused on potential abnormalities in the bony anatomy of the coccyx as a cause of coccydynia. Compared with healthy persons, patients with coccydynia have a more ventrally curved coccyx and a lower prevalence of the sacrococcygeal joint fusion.^8^ Females with coccydynia have a greater prevalence of a bony spicule projection from the terminal coccygeal segment than healthy females, whereas affected males have a greater prevalence of intercoccygeal subluxation.^8^

Histological findings of the coccygeal pad included hyperkeratosis and epidermal hyperplasia in 91% of patients and marked thickening of the dermis due to the proliferation of collagen bundles in 100% of patients.^5^ In the present case, the aforementioned histological characteristics were also noted.

Concerning treatment of the coccygeal pad, 35 of the 42 patients underwent resection of skin nodules.^5^ In 16 of these cases, coccygeal bone resection was performed. However, the recurrence rate is unclear. Posterior hernia of the rectum after total coccygectomy is a rare complication; as far as we know, only 2 cases have been reported.^9^^,^^10^ According to Garcia et al,^9^ the cause of the complication was failure to repair the musculoaponeurotic raphe. The pelvic diaphragm should be repaired by suturing the separated structures of the coccyx, as well as the gluteus maximus muscle, to the posterior sacral aponeurosis.^9^ In the present case, anterior dislocation of the coccyx was marked, suggesting its involvement in nodule development. Therefore, the prominence of the coccyx was flatly resected, and we repaired the pelvic diaphragm. There has been no recurrence and no complication.

In the present case, the nodule, 7 × 4 cm in diameter, was resected and the defect could be closed tension freely. Therefore, there was no postoperative complication and no recurrence.

Hashimoto et al^5^ reported that the skin nodule was operated by using elliptical excision with primary midline closure in 35 cases, which were 1 to 4 cm in short diameter and 2 to 8 cm in long diameter. No reports described dehiscence and recurrence.

In the case of the larger size of the coccygeal pad that cannot be closed tension freely, it might be better to use asymmetric or oblique closure techniques, as well as flap techniques such as rhomboid flaps, VY-plasty, and Z-plasty as used in the treatment of pilonidal disease.^11^

Phyma lesions to be differentiated from the coccygeal pad include human tail,^12^^,^^13^ pilonidal sinus,^14^ and bursitis.^14^ Human tail is a congenital disease, whereas the coccygeal pad is an acquired disease; therefore, each disease can be differentiated on the basis of the patient's medical history. Histologically, pilonidal sinus is an inflammatory granulomatous lesion involving hair. However, the coccygeal pad can be clinically differentiated because there is no fistula or hair. Bursitis is characterized by the puncture-related excretion of serous fluid and a cystoma structure on imaging, but the coccygeal pad does not show these features, and thus differentiation is possible.

The coccygeal pad induces an elastic-hard nodule in the coccygeal area as an acquired lesion. Histologically, hyperkeratosis, epidermal hyperplasia, and proliferation of collagen bundles in the dermis are observed. Radiologically, an anterior flexed coccyx is noted. These are key points for diagnosis. Plastic surgeons should recognize this as a disease to be differentiated from gluteal skin tumors.

## References

[1] Ota J, Morimoto Y, Naito Y, Kawatsu T (1985). Tylosis-like eruption in the sacral area. Hifu.

[2] Murasawa S, Aoki F, Yoshida T, Saijo M, Nakatani Y (1993). “Coccygeal pad” a new term for specific cutaneous lesion. Jpn J Plast Surg.

[3] Harada R, Kawakubo Y (1990). Tylosis-like eruption in the sacral area. Rinsho Hifuka.

[4] Teranisi H, Kanda R, Hata S (1990). An adult case of tylosis-like plaque on the sacro-coccygeal area. Hifu.

[5] Hashimoto I, Shono Y, Ishida S, Nakanishi H (2013). Developmental mechanism of juvenile coccygeal fibrosis (so-called coccygeal pad). J Dermatol.

[6] Nakamura A, Inoue Y, Ishihara T, Matsunaga W, Ono T (1995). Acquired coccygeal nodule due to repeated stimulation by a bicycle saddle. J Dermatol.

[7] Simpson J (1859). Clinical lectures on the diseases of women. Lecture XVII: coccydynia and diseases and deformities of the coccyx. Med Times Gazette.

[8] Woon JTK, Maigne J-K, Perumal V, Stringer MD (2013). Magnetic resonance imaging morphology and morphometry of the coccyx in coccydynia. Spine.

[9] Garcia FJ, Franco JD, Marquez R, Martinez JA, Medina J (1998). Posteriol hernia of the rectum after coccygectomy. Eur J Surg.

[10] Balkenende U, Van Leeuwen BP, Ginai AZ (1996). Hernia through a scar on the posterior rectal wall. Eur J Surg.

[11] Dass TA, Zaz M, Rather A, Bari S (2012). Elliptical excision with midline primary closure versus rhomboid excision with Limberg flap reconstruction in sacrococcygeal pilonidal disease: a prospective, randomized study. Indian J Surg.

[12] Dubrow TJ, Wackym PA, Lesavoy MA (1988). Detailing the human tail. Ann Plast Surg.

[13] Fara M, Smahel J (1973). Human tail. Acta Chir Plast.

[14] Bradley L (2010). Pilonidal sinus disease: a review, part 1. J Wound Care.

